# Natural history of alpha mannosidosis a longitudinal study

**DOI:** 10.1186/1750-1172-8-88

**Published:** 2013-06-20

**Authors:** Michael Beck, Klaus J Olsen, James E Wraith, Jiri Zeman, Jean-Claude Michalski, Paul Saftig, Jens Fogh, Dag Malm

**Affiliations:** 1 Center for Pediatric and Adolescent Medicine, University Medical Center, Langenbeckstraße 1,55131 Mainz, Germany; 2 Larix ApS, Tempovej 44,1, 2750, Ballerup, Denmark; 3 Manchester Academic Health Science Centre, Central Manchester University Hospitals NHS Foundation Trust, St Mary’s Hospital, Oxford Road, Manchester, M13 9WL, United Kingdom; 4 Department of Pediatrics, First Faculty of Medicine, Charles University, Ke Karlovu 2, Prague 2 120 00 Czech Republic; 5 Unite’ Mixte de Recherche CNRS/USTL 8576-UGSF, Unité de Glycobiologie Structurale et Fonctionnelle, IFR 147, Bat C9, Université des Sciences et Technologies de Lille, 59655 Villeneuve d’Ascq Cedex, France; 6 Department of Biochemistry, Christian-Albrechts-Universität Kiel, Olshausenstr. 40, 24098 Kiel, Germany; 7 Zymenex A/S, Roskildevej 12 C, 3400 Hillerød, Denmark; 8 The Tromsø Centre of Internal Medicine (TIS as), House of Health, Sjøgata 31/33, NO9008 Tromsø, Norway

**Keywords:** Alpha-mannosidosis, Natural history, Oligosaccharidosis, Oligosaccharides, Survey study

## Abstract

**Background:**

Alpha-Mannosidosis is a rare lysosomal storage disorder, caused by the deficiency of the enzyme alpha-Mannosidase. Clinically it is characterized by hearing impairment, skeletal and neurological abnormalities and mental retardation. In order to characterize the clinical features and disease progression of patients affected by alpha-Mannosidosis, a survey study was conducted. 43 patients from 4 European countries participated in this longitudinal study. Age range of the participants was 3 to 42 years. For each patient a medical history, complete physical and neurological examination, joint range of motion and assessment of physical endurance and of lung function were completed. In addition, serum and urinary oligosaccharide levels were analysed.

**Methods:**

In this multicenter longitudinal study clinical data of 43 alpha-Mannosidosis patients were collected. In addition to objective clinical measurements biochemical assays were performed.

**Results:**

Data analysis revealed a wide spectrum of clinical presentation regarding the severity and disease progression. Most clinical abnormalities were observed in the musculoskeletal and neurological system. All patients showed mental retardation and hearing loss from early childhood. An impairment in physical endurance was revealed by the 6-minute walk and 3-minute stair stair climb tests. There was only slight progression of a few clinical findings: Psychiatric troubles in both groups essentially, and respiratory dysfunction under 18 years. The serum and urinary oligosaccharide levels were increased in all affected individuals and correlated well with the 6-minute walk and 3-minute stair climb test results.

**Conclusions:**

This study confirms that alpha-Mannosidosis is a very heterogeneous disorder regarding both, disease severity and progression. As it has been shown that Mannosidosis patients are able to perform lung function tests and the 6MWT and stair-climb test, these clinical parameters apparently can be used as clinical endpoints for clinical trials. Oligosaccharide levels appeared correlated with functional testing and may serve as biomarkers of disease severity, progression and response to treatment.

**Trial registration:**

ClinicalTrials.gov Identifier = NCT00498420 and EuropeanCommission FP VI contract LHSM-CT-2006-018692.

## Background

Alpha-mannosidosis (OMIM 248500) is a rare lysosomal storage disorder caused by the deficiency of alpha-Mannosidase (MAN2B1, EC 3.2.1.24), a lysosomal enzyme responsible for the degradation of N-linked oligosaccharides. Alpha-mannosidosis is a progressive disorder, and characteristic features include mental retardation, coarse facial appearance, hearing loss, skeletal deformities, central nervous system involvement and immune defects. Based on severity two distinct phenotypes of alpha-Mannosidosis have been described: A severe form (type I) with hepatomegaly and early death caused by severe infections [1], and an attenuated (mild) form (type II) with hearing loss, mental retardation and slow progression with survival into adulthood [2]. A further classification into a mild, moderate and severe type of alpha-Mannosidosis seems to be questionable as the clinical presentation varies considerably [3]. In this paper the term “severe form” (type I) and “mild form” (type II) are used.

Type I alpha-Mannosidosis leads to early death, primarily caused by the involvement of the central nervous system and recurrent infections. In patients affected by the mild form first symptoms such as hearing loss and skeletal abnormalities are often seen before the age of 10 years; later ataxia and mental retardation become more and more evident. In alpha-Mannosidosis broad heterogeneity is seen not only in the clinical manifestations, but also in the spectrum of mutations of the alpha-Mannosidase gene (MAN2B1) that is located on chromosome 19p13.2. Currently, 125 different disease-causing mutations have been identified [4]. The prevalence of alpha-Mannosidosis is not exactly known, Meikle et al. have calculated a prevalence of 1 case in 1 million live births [5], a similar prevalence was observed in the Netherlands [6].

There are many reviews extensively describing the clinical manifestation of the lysosomal storage disorder alpha-Mannosidosis; a longitudinal study, however, evaluating the progression of the disease in a greater number of patients is lacking. Furthermore, the clinical reports that have been published so far have been based on a small number of patients and have not included quantitative measurements of clinical features. Therefore, the EU consortium HUE-MAN (Towards the Development of an Effective Enzyme Replacement Therapy for Human Alpha-Mannosidosis) was established in order to conduct a survey of the natural history study of the human disease alpha-Mannosidosis [7]. The purpose of this study was also to define possible clinical endpoints for future clinical trials. In addition, clinical phenotypes of selected European patients have been determined and important medical data such as the results of clinical and neurological investigations, ophthalmological and hearing examinations, lung and heart function and measurement of general endurance using the 6-minute walk test (6MWT) and 3-minute stair climb test (3MSCT) were collected.

## Methods

### Clinical evaluation

The aim of this multicenter, multinational prospective study was to evaluate clinical and surrogate parameters known to be affected in alpha-Mannosidosis patients. The design of this observational study was open, non randomized with several objective clinical measurements, laboratory measurements and some investigator evaluated parameters. In addition, some patient assessed measurements (Quality of Life and Health Questionnaires) were collected. Informed consent was obtained prior to any study related activity and the subject, or the subject’s legal guardian, provided consent using the Ethics Committee (EC)-approved consent form and with compliance to local and European Union regulations, International Conference on Harmonization (ICH) and Good Clinical Practice (GCP) standards.

Only patients with the mild form (type II) were entered into this study if they had a documented deficiency of serum or leukocyte acid alpha-Mannosidase enzyme activity level. Patients had to be excluded from the study if they had received a bone marrow transplantation or had used an investigational drug within 30 days prior to study enrollment. Known medical conditions or serious intercurrent illness that could significantly interfere with study compliance were further exclusion criteria. In all subjects, clinical symptoms (with special emphasis on hearing difficulties and visual impairment) and history of infections, surgical procedures, hospitalizations and use of medication were recorded. The physical examination included vital signs, height and weight, evaluation for heart murmur, and for liver and spleen size.

As a measure of general endurance the 6-minute walk test (6MWT) was conducted at baseline and at visit 12 and 24 months [8]. The values are given as LOCF = *Last Observation Carried Forward*, that means that for patients who have missing data, the last previous data point which was actually recorded is carried forward. The purpose of this is to get a mean value which is not influenced by the large fluctuations in the means that may occur because of the different levels for the patients. Because there are no published normal values of the 6MWT covering the full age range, the published references were combined in order to overcome this difficulty. For this purpose, a predictive model of the 6MWT outcomes was derived from the models published by Geiger et al. [9], Gibbons et al. [10] and Enright et al. [11]. For the late adolescence, an obvious adjustment to the Geiger model was introduced. In order to join the three models, linear interpolation was applied where the age ranges of the models met. This procedure resulted in a combined predictive 6MWT model that could be used across the full age range. Sensitivity analyses confirmed the suitability of the proposed model.

In the 3-minute stair climb that was conceived on the basis of a combination of published tests [12,13], patients were instructed to climb as many steps as possible in a 3-minute period.

Joint range of motion encompassing passive flexion and extension at the shoulders, elbows, and knees was measured with a goniometer utilizing standard technique. For assessment of heart function a standard 12-lead electrocardiogram (ECG) was performed. Interpretation of the ECG included assessment of heart rate, cardiac rhythm, intervals, axis and conduction defects. Echocardiograms were optional, interpretation included assessment of the size of the chambers and pumping function.

Forced vital capacity (FVC), forced expiratory volume (FEV1) and peak flow were measured in accordance with American Thoracic Society (ATS) standards [14]. The range of FVC was expressed as percentage of expected depending on age, size and sex.

Ophthalmological assessments included slit lamp examination of the cornea and lens, best corrected visual acuity (in dp) and examination of the retina of both eyes at baseline, at 12 month and at 24 months. All three were evaluated and assessed as *Normal* or *Abnormal* and the abnormal were classified as *Non Clinically Significant* or *Clinically significant* for each eye.

Hearing was tested by pure-tone audiometry for air and bone conduction in the conventional frequency range and for air-conduction in the extended high frequency range.

For assessment of activities of daily living, severity of pain, and extent of disability the Health Assessment Questionnaire (HAQ) was used for subjects older than 18 years of age [15]. For subjects ≤ 18 years of age the Childhood Health Assessment Questionnaire (CHAQ) was completed by the caregivers [16].

### Oligosaccharide analysis

#### Serum

Serum samples used in this study were stored at −20°C. Control serum samples were obtained from healthy volunteers within the department.

250 μL of serum were mixed with 1 μg of a disaccharide (Man(β1-4)GlcNAc) which served as an internal standard. The glycans were purified on a C18-Sep-Pak (Waters Ltd) and on a column of 150 mg of nonporous graphitized carbon (Alltech, Deerfield, IL, USA). After conditioning, the C18-Sep-Pak by sequential washing with methanol (5 mL), and 5% acetic acid (10 mL), the sample was loaded onto the Sep-Pak and the glycans were eluted with 3 mL of 5% acetic acid. The glycans were then desalted on a column of 150 mg of nonporous graphitized carbon (Alltech, Deerfield, IL, USA). The column was sequentially washed with 5 mL methanol and 10 mL 0.1% v/v TFA. The glycans were applied to the column and washed with 15 mL of 0.1% v/v TFA. The elution of the glycans was conducted with the application of 5 mL of 25% v/v acetonitrile in water containing 0.1% v/v TFA. The fractions were freeze-dried.

Glycans were derivatized with 2-aminobenzamide as previously described, with minor modifications [17]. The freeze-dried glycans were dissolved in 100 μL of a solution (freshly prepared by mixing 64 mg of sodium cyanoborohydride, 41 mg of 2-aminobenzamide, 700 μl of dimethylsulfoxide, and 300 μl of acetic acid). The reaction mixture was stirred for 2 h at 80°C. To remove the excess of reagents, 500 μL of 75 and 85% methanol was added in succession to the reaction mixture and evaporated. After addition of 2 mL of water, the pH of the solution was adjusted to 10 with diluted ammonia solution and the excess of reagents was extracted with 500 μL of chloroform (five times). The aqueous phase was neutralized with dilute acetic acid prior to lyophilization. Finally, the derivatized glycans were further purified on a Sep-Pak C_18_ (Waters, Saint-Quentin en Yvelines, France). The Sep-Pak C_18_ was conditioned with methanol (5 mL) and water (10 mL). The derivatized glycans dissolved in water were applied on the cartridge, washed with 15 mL of water and eluted with 3 mL of 25% acetonitrile in water. Acetonitrile was evaporated under a stream of nitrogen and the 2-aminobenzamide derivatized glycans were freeze-dried.

The 2-aminobenzamide labeled glycans were loaded on a Shodex Asahipak NH2P-50 column (5 μm; 4.6 × 250 mm; VWR). The mobile phases were acetonitrile (solvent A) and water (solvent B). The column was equilibrated with 85% A. After injection, isocratic conditions were applied for 15 min with 85% A, followed by a gradient with the following conditions: (step 1) 85-80% A for 15 min, linear gradient; (step 2) 80-50% A for 60 min, linear gradient; (step 3) 50% A for 20 min. The flow rate was 0.7 mL/min. 2-aminobenzamide derivatized glycans were detected on a Dionex RF 2000 fluorescence detector at an excitation of 350 nm and an emission wavelength of 450 nm.

#### Urine

Samples used in this study were stored at - 20°C. Control urine samples were obtained from healthy volunteers within the department.

Urine (0.207 μmol creatinine equivalents) was mixed with 2.608 nmol of a disaccharide (Man(β1-4)GlcNAc) which served as an internal standard and freeze-dried. Glycans were derivatized with the 2-aminobenzamide as previously described, with minor modifications [17]. The freeze-dried glycans were dissolved in 100 μL of a solution (freshly prepared by mixing 64 mg of sodium cyanoborohydride, 41 mg of 2-aminobenzamide, 700 μl of dimethylsulfoxide, and 300 μl of acetic acid). The reaction mixture was stirred for 2 h at 80°C. To remove the excess of reagents, 500 μL of 75 and 85% methanol was added in succession to the reaction mixture and evaporated. After addition of 2 mL of water, the pH of the solution was adjusted to 10 with diluted ammonia solution and the excess of reagents was extracted with 500 μL of chloroform (five times). The aqueous phase was neutralized with dilute acetic acid prior to lyophilization. Finally, the derivatized glycans were further purified on a Sep-Pak C_18_ (Waters, Saint-Quentin en Yvelines, France). The Sep-Pak C_18_ was conditioned with methanol (5 mL) and water (10 mL). The derivatized glycans dissolved in water were applied on the cartridge, washed with 15 mL of water and eluted with 3 mL of 25% acetonitrile in water. Acetonitrile was evaporated under a stream of nitrogen and the 2-aminobenzamide derivatized glycans were freeze-dried.

The 2-aminobenzamide labeled glycans were loaded on a Shodex Asahipak NH2P-50 column (5 μm; 4.6 × 250 mm; VWR). The mobile phases were acetonitrile (solvent A) and water (solvent B). The column was equilibrated with 85% A. After injection, isocratic conditions were applied for 15 min with 85% A, followed by a gradient with the following conditions: (step 1) 85-80% A for 15 min, linear gradient; (step 2) 80-50% A for 60 min, linear gradient; (step 3) 50% A for 20 min. The flow rate was 0.7 mL/min. 2-aminobenzamide derivatized glycans were detected on a Dionex RF 2000 fluorescence detector at an excitation of 350 nm and an emission wavelength of 450 nm. The mass of these glycans were determined using MALDI-TOF-MS.

#### Matrix-assisted laser desorption/ionization time-of-flight (MALDI-TOF) mass spectrometry

MALDI-MS experiments were carried out on Voyager Elite DE-STR Pro instrument (PersSeptive Biosystem, Framingham, MA) equipped with a pulsed nitrogen laser (337 nm) and a gridless delayed extraction ion source. The spectrometer was operated in positive reflectron mode by delayed extraction with an accelerating voltage of 20 kV and a pulse delay time of 200 nsec and a grid voltage of 66%. All spectra shown represent accumulated spectra obtained by 400–500 laser shots. Sample was prepared by mixing a 1 μL aliquot (5–10 pmoles) with 1 μL of matrix solution on the MALDI sample plate. The matrix solution was prepared by saturating methanol–water (1:1) with 2,5 dihydroxybenzoic acid (DHB) (10 mg/mL).

## Results

### Demographics and body measurements

Fourty five patients from 4 centers (Children’s Hospital, University of Mainz, Germany; Department of Medicine, University Hospital of Tromsoe, Norway; Dept. of Genetic Medicine, St. Mary’s Hospital, Manchester UK; and the Department of Pediatrics, Charles University, Prague, Czech Republic) could be screened for this study; 31 were males and 14 were females. The age range was 1.4 – 42.1 years, mean age was 19.8 years. 22 were under the age of 18 years and 23 patients 18 years or older at the time of enrolment. 43 patients completed the 2 years, two children discontinued because of non-compliance.

**Figure 1:**
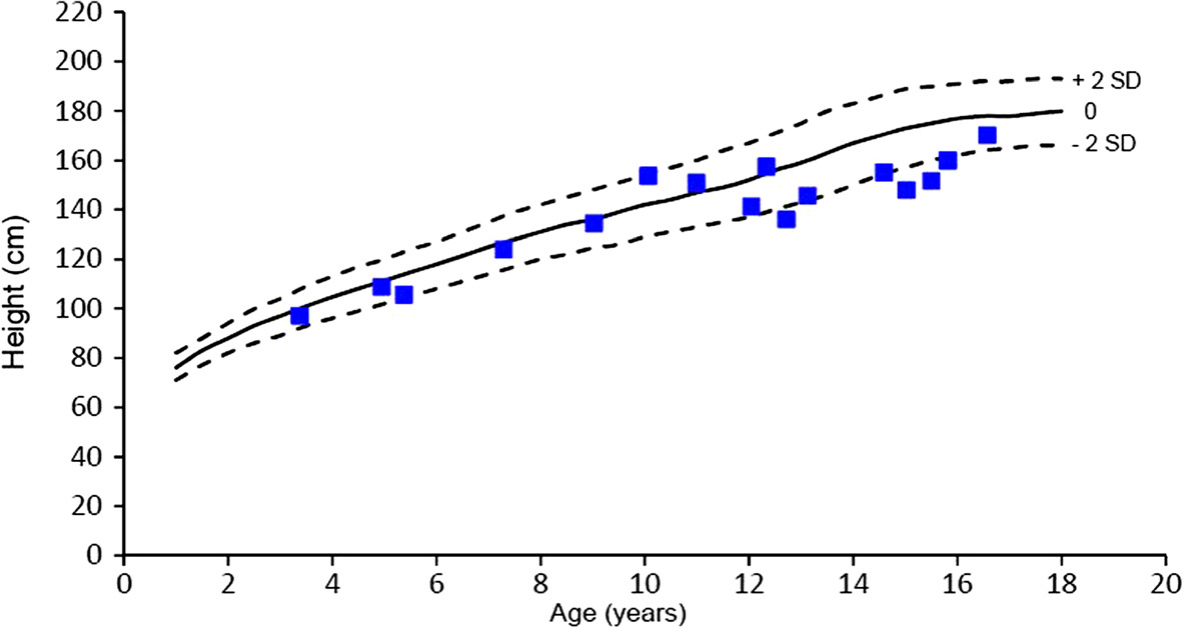
**Height of males with Alpha-Mannosidosis.** Outer lines indicate - 2 SD and + 2 SD.

**Figure 2:**
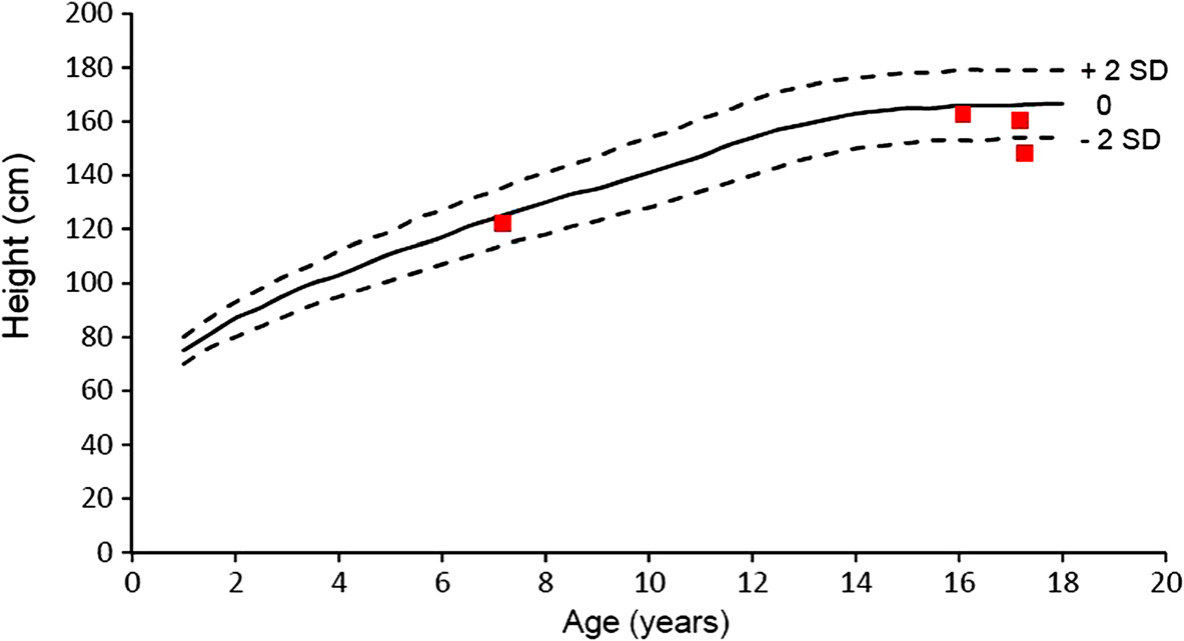
**Height of females with alpha-mannosidosis.** Outer lines indicate - 2 SD and + 2 SD.

Figures 1 and 2 show the absolute height of males and females at baseline, compared to published growth curves for normal individuals from Northern European countries [18]. Only 4 children (3 boys and 1 girl) had an height below the curve of the double standard deviation. Mean height of the adult patients was 162 ± 9 cm SD with a broad range from 145 to 179 cm.

**Figure 3:**
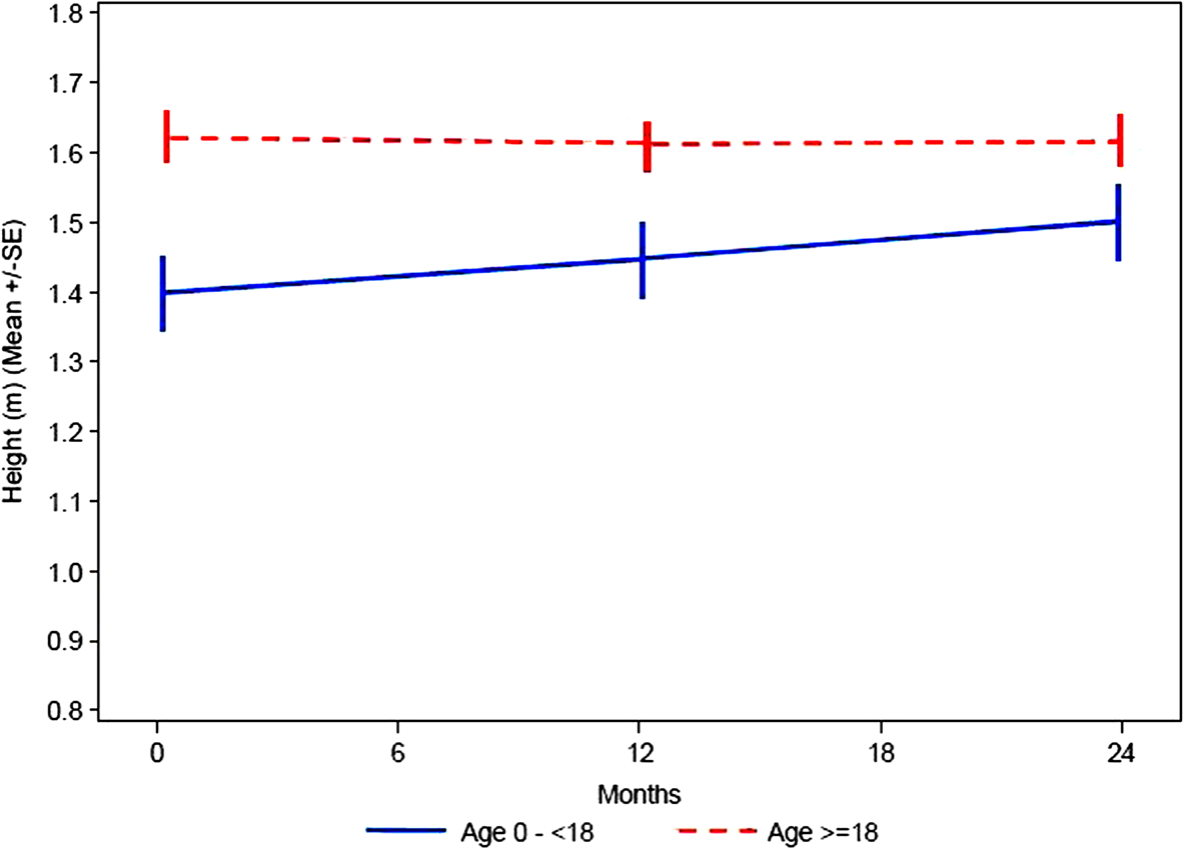
Change of height of children and adults over time.

There is a natural increase in height of patients in the age group < 18 years as the majority of patients are in a growth period (Figure 3).

Most of the patients in the younger age have an overall normal BMI, there are two girls, 16 and 17 years old, who are overweight (BMI = 28.2 and 25.4, respectively). In the adult group five patients (3 males and 2 females) are overweight with a BMI greater than 24.0; furthermore, in this group there are two females (age 27 and 32) who show severe obesity with a BMI of 39 and 33, respectively.

### Clinical assessments

All patients had a physical examination performed at baseline, 12 months and at 24 months. Due to an unexpected early termination of the study several patients could not complete visit 3 after 24 months.

In the general examination, all patients (children and adults) had any abnormal clinical findings such as coarse facial features (13 of 43), ear infections (8 of 43), heart murmur (9 of 43) and skeletal deformities such as kyphoscoliosis and pectus carinatum.

Data from the ophthalmological investigations showed that 2 patients (age 14 and 22 years) had corneal clouding, 2 patients (age 39 and 30 years) had a cataract and 2 patients (age 15 and 34 years) were almost blind (vision = 6/95 at the better-seeing eye) due to retinal pigmentary degeneration. In one patient (25 years) a pale papilla was seen.

From Table 1 it can be seen that the baseline visual acuity is approximately the same for both age groups. Over the observation period, there is only a insignificant deterioration in visual acuity.

**Table 1:** Visual acuity (best corrected, best seeing eye) of both age groups

	**Normal vision (6/6 - ≥ 6/12)**	**Low vision (≤ 6/12 - ≥ 6/120)**
***Age Group***	**N**	**N**
Age < 18 Years (N = 20)	18	2
Age ≥ 18 Years (N = 23)	20	3

**Figure 4:**
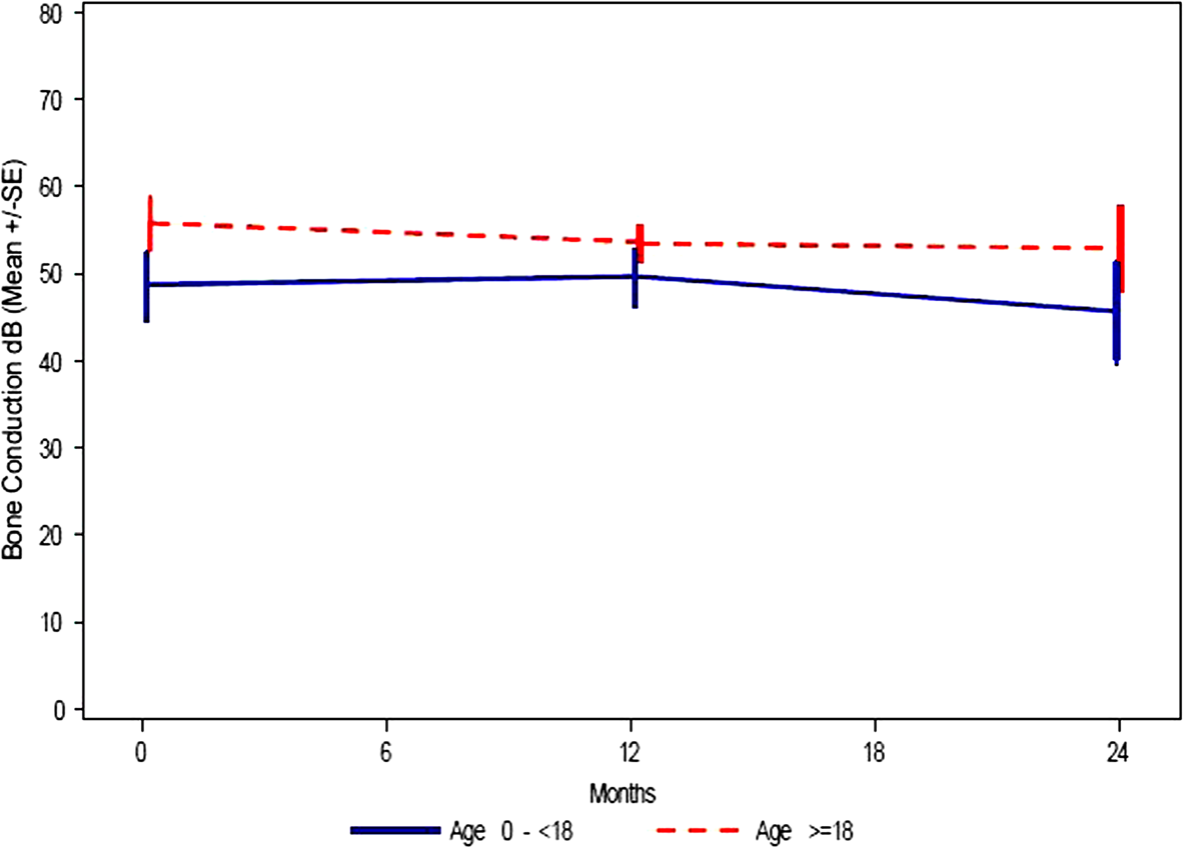
**Hearing testing - bone conduction (dB HL).** Mean ± SE (= 1 Standard deviation).

**Figure 5:**
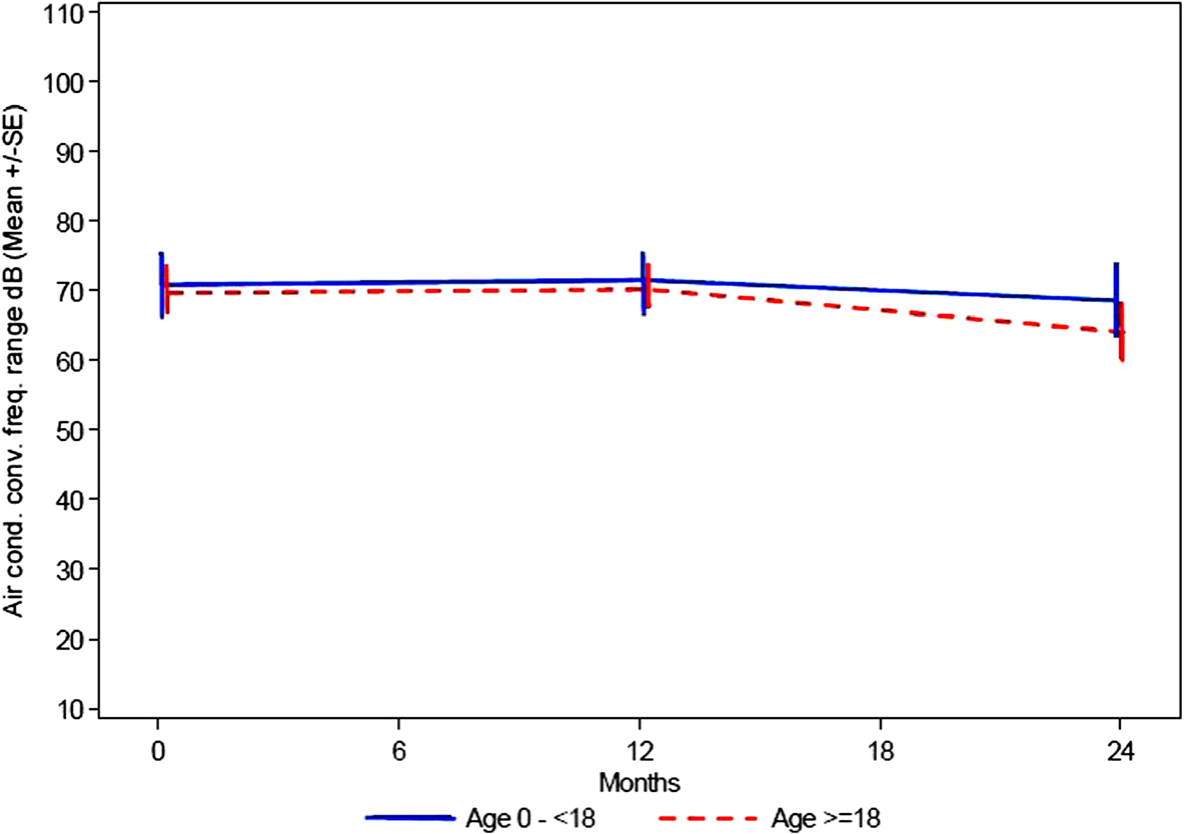
Hearing testing - air conduction: conventional frequency range (dB HL) mean ± SE (= 1 Standard deviation).

**Figure 6:**
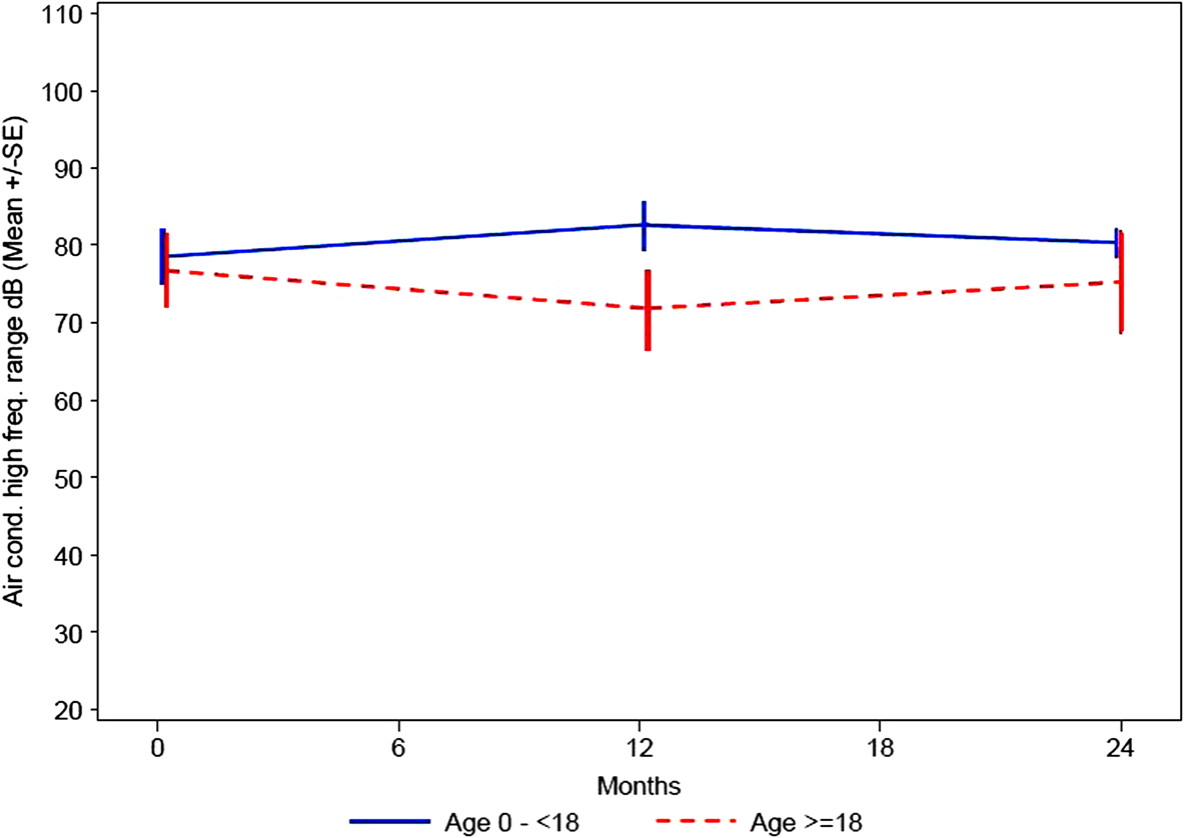
Hearing testing air conduction: high frequency range (dB HL) Mean ± SE (= 1 Standard deviation).

All patients who were able to cooperate had an assessment of their hearing performed at baseline, at 12 months and at 24 months. The assessment included: Bone conduction, air conduction (conventional frequency) and air conduction (high frequency). All patients over the age of 3 years had a significant hearing loss and had to wear hearing aids. Data of the hearing tests, expressed as decibel, show that baseline values are quite similar between the two age groups (Figures 4, 5, 6).

The data also seem to indicate a minor change of hearing loss over the first part of the observation period which also is similar between the groups. For the second part of the observation period from 12 to 24 months there seems to be a slight improvement in hearing. Missing data limits a more detailed interpretation of the results.

For the cardiovascular system 24% of the patients under the age 18 years and 17% of patients age 18 or over presented abnormal findings. In the majority of cases they were clinically not significant as exemplified as “murmur” on cardiac auscultation. Five patients had an abnormal echocardiogram: The findings were given as valvular insufficiency or decreased ventricular ejection fraction. Two patients (one in each age group) had an abnormal ECG that was assessed as clinically significant; more findings were not reported.

The musculoskeletal area showed typical abnormal findings for this disease such as macrocephaly, contractures, scoliosis, genua valga, hip dysplasia and deformities of feet. In the younger age group (patients < 18 years) 62% of the patients had abnormal findings for this body area. It seems to be stable over the observation period, which would also be expected. In the older patients pathological signs and symptoms of the musculoskeletal system were observed at an even higher rate than in the younger age group: In this population 92% of the patients had abnormal findings for this body area at baseline. After two years 94% of adult patients had abnormal findings.

Neurologically 71% of the 20 patients under the age of 18 years showed abnormal signs and symptoms at baseline, whereby the main clinical sign was ataxia that was seen in 12 of the patients. Further findings included dysarthria, dysmetria, and mental retardation. A similar rate of pathological neurological findings (79%) was seen in the older patients. Over the observation period this did not change significantly, in either the younger or older age group.

Psychologically, 52% of the patients had abnormal findings at baseline. Despite a decrease at visit 2 in the percentage of patients with abnormal findings (e.g. psychotic disorder) there was a significant increase at visit 3 with 79% of the patients showing abnormal findings.

### 6-minute walk test and 3-minute stair climb test

All patients were expected to perform a 6-minute walk test (6MWT) at baseline, at 12 months and at 24 months. However, as the youngest patient was not cooperating, only 19 of 20 patients under the age of 18 carried out the test at baseline. Following amendment 1 some patients did not have the opportunity to complete either the amended 18 months test (due to the timing of the amendment) or the 24 months test (because of the early stop of the study). Hence the number of patients completing the projected tests differ considerably between time points.

As shown in Table 2 the distance that could be walked within 6 minutes at baseline ranged from 60,0 to 470,0 m with a mean value of 341,1m (SD = 104,9 m) in the younger age group, and from 69,0 m to 521,3 m with a mean value of 324,7 m (SD = 118,1 m) in the older group.

**Table 2:** Results of the 6-min walk test

		**N**	**Mean**	**Std.**	**Min**	**Max**
**Age 0 - < 18 years**						
Distance (m)						
	Baseline	19	341.1	104.9	60.0	470.0
	12 Months	19	340.0	106.3	182.3	540.0
	18 Months	10	331.6	128.9	94.0	478.0
	24 Months	13	393.2	142.3	164.0	770.0
**Age ≥ 18 years**						
Distance (m)						
	Baseline	23	324.7	118.1	69.0	521.3
	12 Months	19	325.4	139.2	64.0	573.0
	18 Months	6	450.5	134.2	254.0	630.0
	24 Months	15	305.8	89.2	105.0	430.0

**Figure 7:**
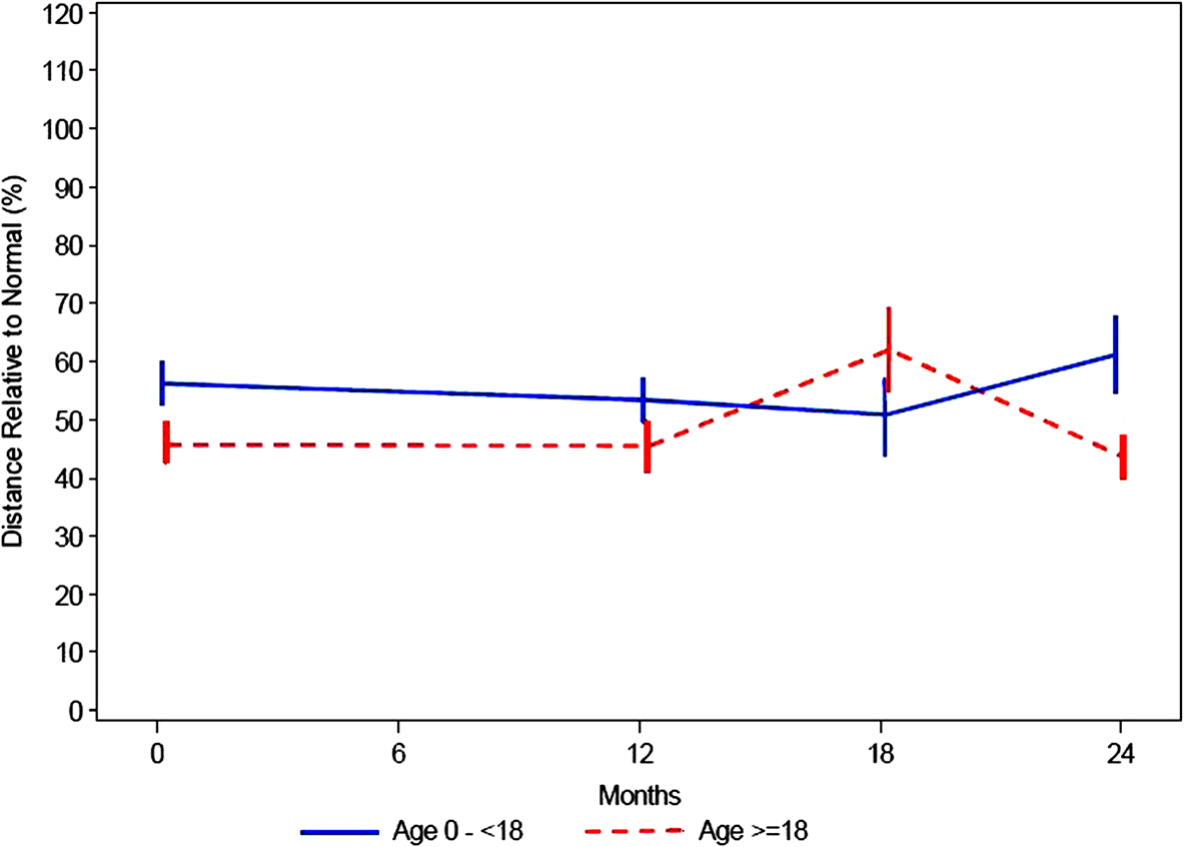
6-minute walk test: percentage of normal ± SE.

In order to compare the outcome of the 6MWT in the patient group with normal subjects, the predictive model of the 6MWT outcomes, published by Geiger et al. [9], Gibbons et al. [10] and Enright et al. [11], was used (see Methods). Calculations by using the Geiger model show that for the patients in the younger patient population the functional capacity is reduced to a value between 50 and 60% of the values for normal patients of the same age (Figure 7). For the older age group (above 18 years of age) the functional capacity for a 6 MWT is further reduced to less than 50% of the functional capacity for normal patients (ie 40 – 45%).

At baseline, the number of stairs that could be climbed was 92–166 stairs with a mean value of 141 (SD = 30) stairs in the younger age group, after 1 year a slight improvement was observed (106–197 stairs, mean value 160, SD = 39 stairs). Patients at the age over 18 years could climb at baseline 22–186 stairs (mean value = 100, SD = 58), they also showed an improvement after one year (43–197 stairs, mean value = 126, SD = 55 stairs). Data of the 2 years visit are not available.

### Lung function tests

Eleven of patients under the age of 18 years and 9 adult patients were able to perform a pulmonary function test at baseline, at 12 months, at 18 months and at 24 months. The assessments included: Forced vital capacity (and percentage of predicted value, depending on age, size and sex = % predicted FVC), forced expiratory volume during first second (and percentage of predicted value = % predicted FEV1) and peak expiratory flow rate. In all measurements there was a significant range, spanning from about 30% to 90% of predicted FVC and FEV1. No patient was dependent on night time ventilation.

**Figure 8:**
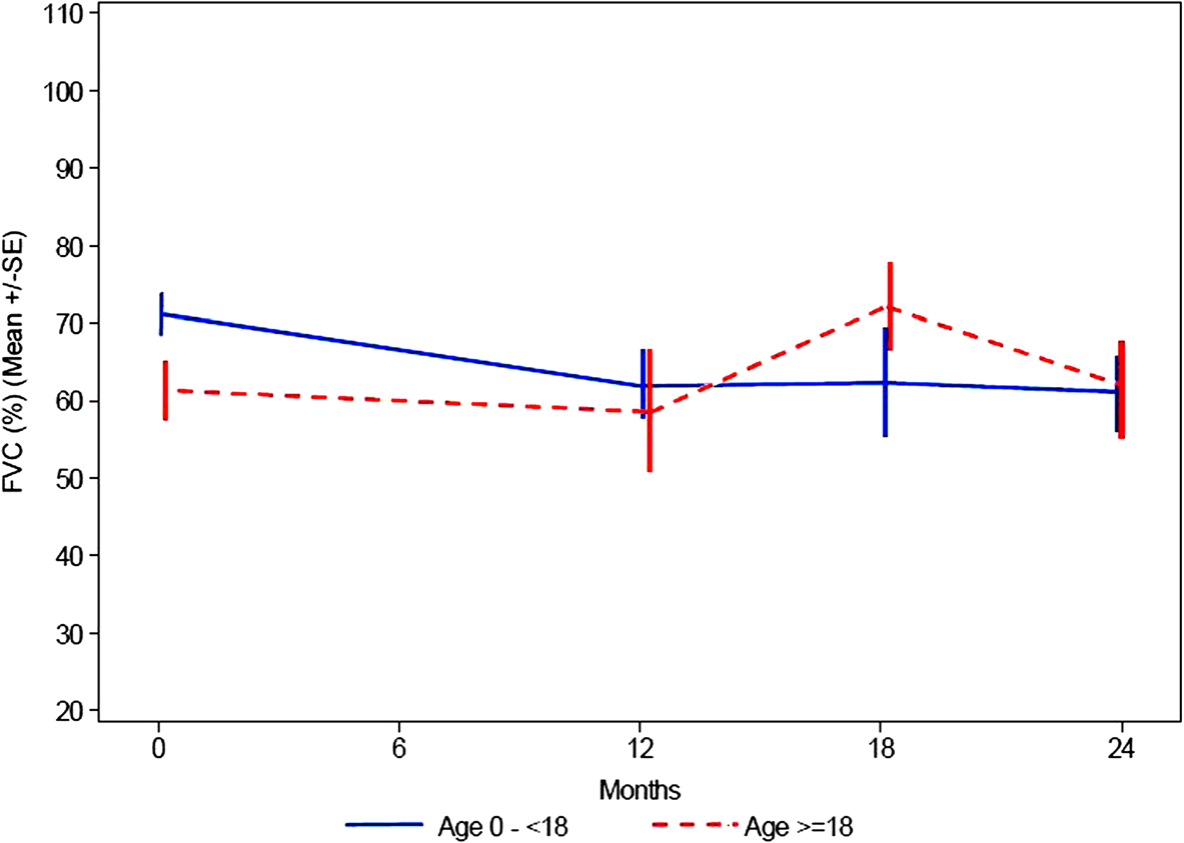
Lung function: range of FVC, expressed as percentage of expected depending on age, size and sex.

In patients under the age of 18 the % predicted FVC is decreasing from a baseline value of 71% to 61% at visit 3 (Figure 8) that clinically is not significant.

**Figure 9:**
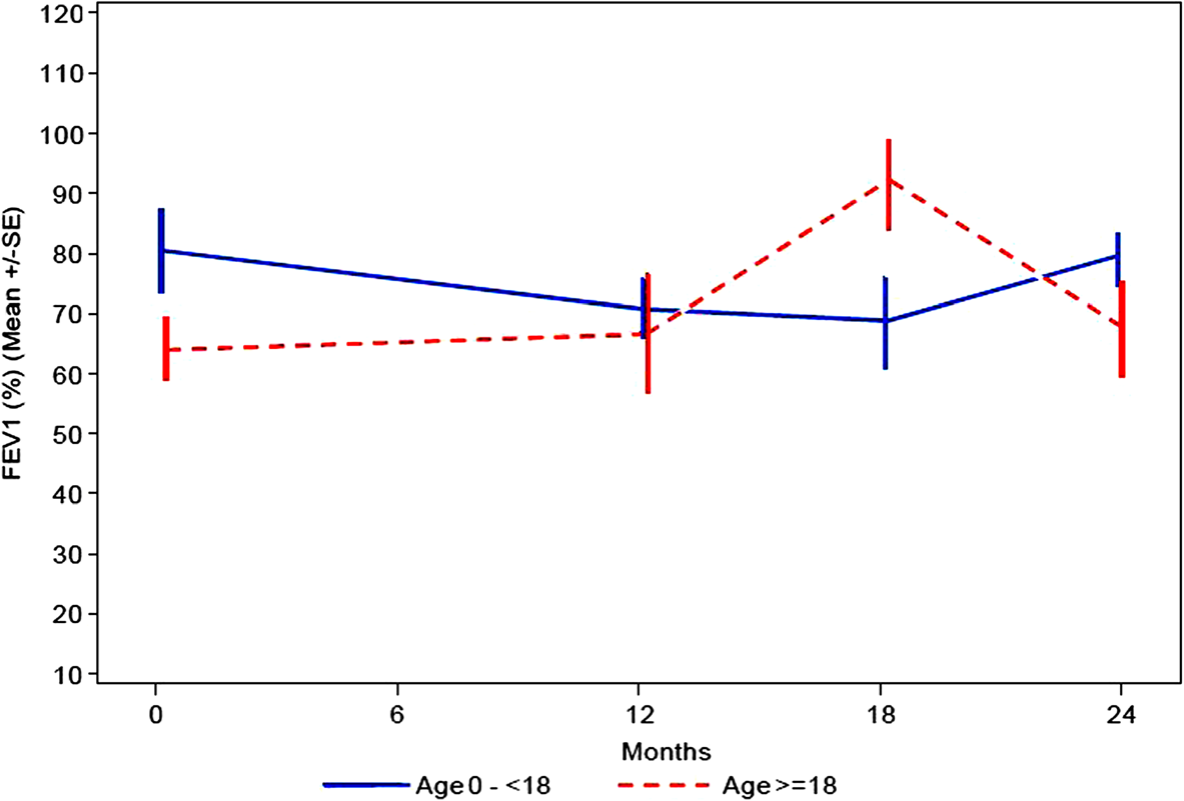
Lung function: percentage of predicted forced expiratory volume during first second.

For % predicted FEV1 the data also show no significant change within 24 months (Figure 9). In the older age group the pulmonary function capacity is similar at baseline and after the 2 years period.

For the older age group the overall picture of the pulmonary function capacity is similar over time; for the younger age group the data seem to indicate that over the observation period there is up to a 10% decrease in pulmonary function, that clinically is not significant.

### Cardiology assessment

All patients had an assessment of their ECG and an overall interpretation of an echocardiogram at baseline, at 12 months and at 24 months. The ECG and echocardiogram were evaluated and assessed as *Normal* or *Abnormal* and the abnormal results were further classified as *Non Clinically Significant* or *Clinically significant*. There are two patients in the older age group and one in the younger age group who present with clinically significant abnormal findings. The findings are given as valvular insufficiency or stenosis or decreased ventricular ejection fraction. These abnormal findings do not change over the observation period.

### Oligosaccharides in serum and urine

**Figure 10:**
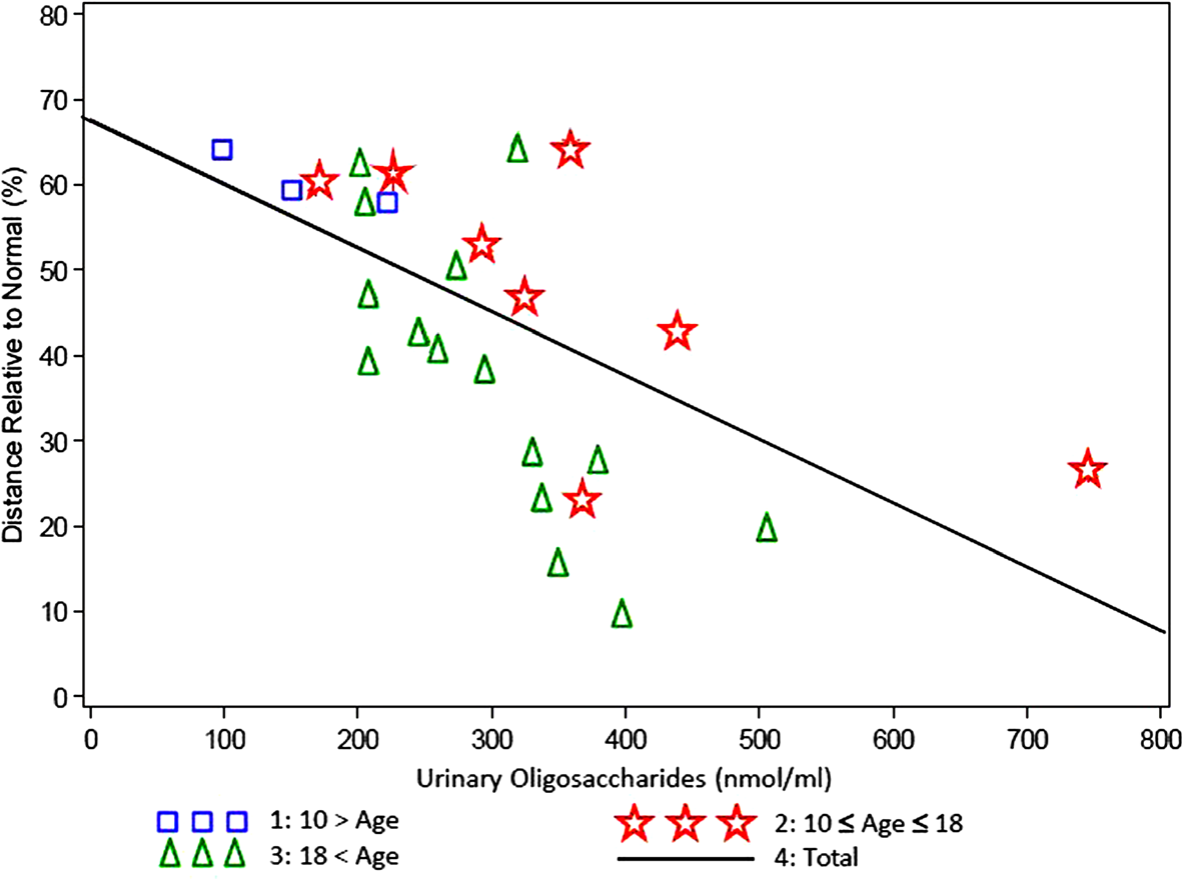
**6 minutes walk test (% of predicted) versus oligosaccharide level in urine by age group.** Correlation Factor = 0,61602, P-Value = 0,0014).

**Figure 11:**
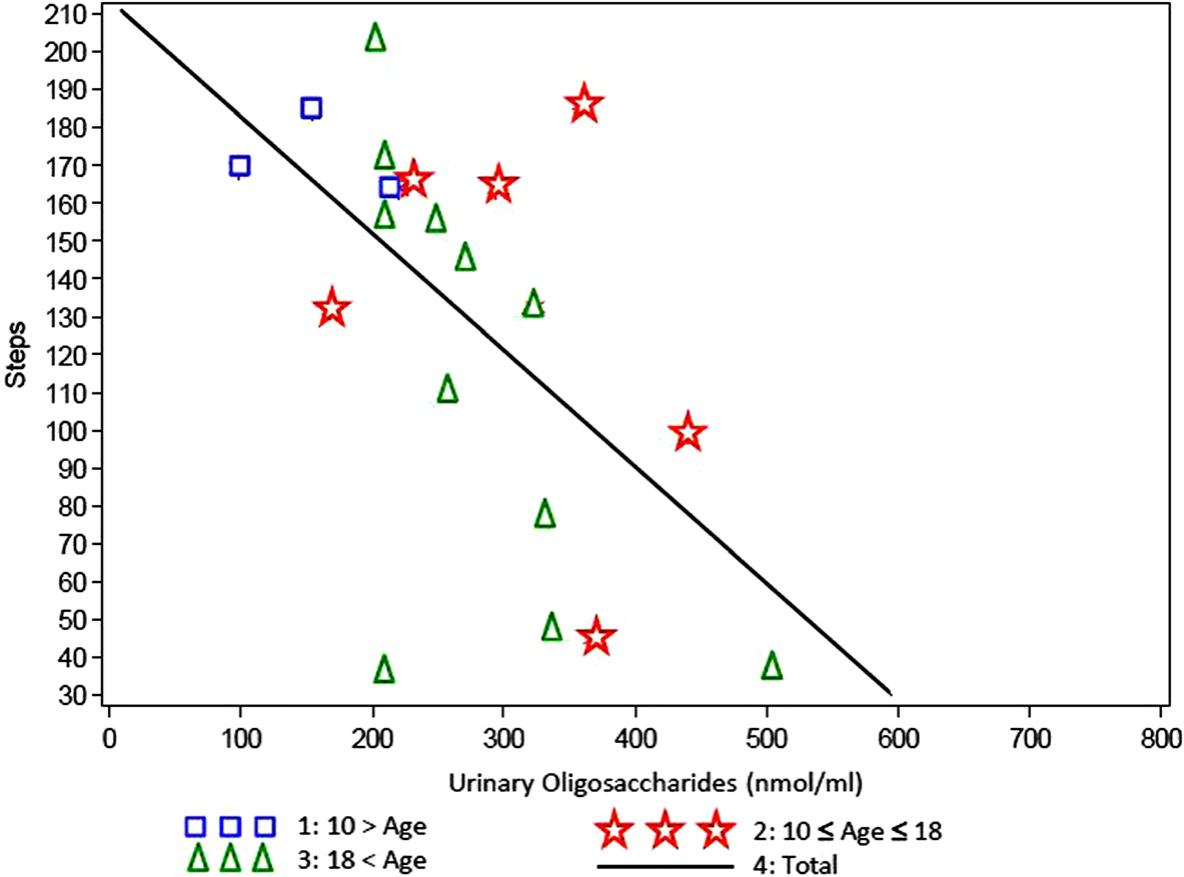
**Minutes stair climb test versus oligosaccharide level in urine by age group.** Correlation Factor = 0,56805, P-Value = 0,0072).

Valid serum oligosaccharide levels were available for 5 subjects under the age of 18 years and from 11 individuals over 18 years. The older patients had higher concentrations (11.05 – 82,45 nmol/ml, mean = 21,49 nmol/ml) than the younger patients (7.16 – 33.49 nmol/ml, mean = 16.33 nmol/ml). In the urine, the oligosaccharide concentration was similar in both age groups, whereby the mean value at the age under 18 years (n = 10) was 323.00 nmol/ml and in adults (n = 15) 280.62 nmol/ml. The 6MWT as an indicator of general endurance was examined as a function of oligosaccharide urine/serum. And as can be seen in Figure 10, low oligosaccharide levels corresponded to a long walking distance and vice versa. Similar results were obtained when the analysis was applied to the 3 minute stair climb test (Figure 11).

### The health assessment questionnaires

The health assessment questionnaires confirm that the patients are dependent to a high degree upon third party assistance. This is not age dependent as it is seen in both age groups. A major difference is the pain score: The VAS scores clearly show that the older age group seem to have more pain than the younger age group (as far as the mentally retarded children could give faithful responses). The reason for this is not clear, but it is likely to represent progressive bone and joint disease.

## Discussion

In the present study that was aimed to establish the range and diversity of clinical features of alpha-Mannosidosis and to define clinical endpoints for future clinical trials, information was collected for 43 affected subjects over the age of 3 years, identified by four centers from four European countries. A total of 45 patients were screened; during the course of the study, however, two patients discontinued because of no-complicance. In addition to clinical investigations, analyses of oligosaccharide concentration in urine and serum and were performed.

This longitudinal survey was also conducted in order to assess the natural history of patients diagnosed with alpha-Mannosidosis and to evaluate short term (24 months) changes in disease parameters. Previously only a few retrospective studies describing the clinical course have been published and these have included only a small number of patients: Yunis et al. analyzed the clinical course of five patients who in their age ranged from 15 to 24 years [19]. The authors pointed out that the first signs and symptoms such as hearing problems, skeletal changes and facial coarsening could be seen from the age of five years; the degree of clinical expression, however, varied from patient to patient, even among siblings. In the brother and sister, described by Ara et al., at the age of 27 and 29 years, respectively, neurological changes such as cerebellar syndrome, hearing loss and mental retardation were the leading clinical signs; except in the sisters’s speech capacity, over a period time of 25 years no progression was observed [20]. Neurodevelopmental assessments including general intelligence, language, visual spatial skills and overall adaptive abilities were performed in three brothers with alpha-Mannosidosis; follow-up studies did not show signs of progressive deterioration of cognitive deficits, except receptive language capability [21].

The data from the physical examination confirm that alpha-Mannosidosis is a complex disorder affecting many organs such as the eyes, ears and the central nervous and the musculoskeletal system. Abnormalities of the musculoskeletal system are one of the clinical findings that affect almost all patients. Whereas in patients under the age of 18 years skeletal deformities were seen in 62%, scoliosis, genua valga, hip dysplasia and joint contractures have been observed in almost all adult patients (92%). A similar frequency of skeletal changes (55 of 59 patients) was described in the review of Chester et al. [22]. A lessening of the bone abnormalities, as described by Yunis et al. [19] could not be confirmed in our study; the severity of of bone deformities did not change over the time. In contrast to the observations of Autio et al. [23] and Yunis et al. [19] a noteworthy growth retardation was not seen in all patients in spite of significant skeletal abnormalities: Only four children had an height below the normal range, in the adults a broad range of height from 145–179 cm was seen. Only a few patients have a BMI above the normal range.

The joint range was measured rigorously, but in a non functional manner, and despite many abnormal findings in the physical examination for the joints, there seemed to be no deterioration in range of joint movement over the observation period.

Ataxia and mental retardation were the prominent neurological findings observed in this study; a progression of these symptoms within 2 years could not be seen. A similar observation was made by Autio et al. who described a merely slow progression of mental retardation in eight patients. These patients had an IQ of 60–80, whereby they scored better in nonverbal tests which can be explained by the speech disturbance due to the hearing impairment [23].

Hearing loss (bone and conductive) is seen in all patients affected by an alpha-Mannosidosis [22,24]. The impaired hearing is predominantly due to sensorineural damage, but because it may result also from early ear infections it represents a mixture of conductive and neurosensory components as it has been seen in this study. As hearing loss appears during early childhood, it is not surprising that in our study both age groups show more or less the same degree of hearing impairment. A significant deterioration within the observational time could not be seen.

Ophthalmological investigations in most patients revealed only minor abnormalities such as slight corneal opacities or cataract and amblyopia. These results are in accordance with the observations of other authors who also have described strabismus, hypermetropia and slight corneal clouding in only a few cases [23,25]. Retinal pigmentary degeneration, leading almost to blinding, has been found in two of our patients; a similar observation was made by Springer et al. in two brothers [26]. In general, however, retinal dystrophy seems to be rare in Mannosidosis patients. There were no significant changes of the ophthalmological findings in our study throughout the observation period.

Besides innocent heart murmur the majority did not have significant cardiac abnormalities. In five patients, however, valvular insufficiency or decreased ventricular ejection fraction was seen by echocardiogram. Two patients had an abnormal ECG that was not specified in detail by the investigator.

Because to our knowledge there is a paucity of data on respiratory function in Mannosidosis patients, lung function tests were performed in the present study. However, not all patients were able to perform the lung function tests because of their mental status. In the younger age group there was a decline of percentage predicted FVC within the observation time of 2 years. The older patients had a lower percentage predicted FVC (61%) at baseline in comparison with the children (Figure 8).

The 6 MWT has not previously been performed in Mannosidosis patients, therefore this method was used to measure the general endurance of these individuals in the present study. However, both the actual distance measured as well as the percentage of normal distance does not show any consistent picture either in the younger or in the older patient age group. Both groups show a significant range, spanning from about 60 to about 500 meters. A number of factors such as parental influence, growth or a training effect may have influenced the 6MWT. A similar consideration applies to the 3 minute stair climb test. There is no clear trend throughout the observation period for either age group probably for similar reasons as stated for the 6 MWT.

Mannosidosis patients excrete high amounts of undegraded oligosacharides that can be detected in their urine. Serum samples showed similar findings. The storage material is composed mainly of tri-, tetra-, and pentasaccharides and probably represents the products of endo-ß-N-acetylglucosaminidase digestion of the sugar chains of high mannose type glycoproteins [27,28]. In our study, an interesting correlation of the 6MWT and oligosaccharide excretion could be observed in both age groups: Patients with a high oligosaccharide excretion have an impaired ability to walk (Figure 10). The same correlation was found of oligosaccharide excretion and the stair climb test (Figure 11). As a relationship of general endurance and urinary oligosaccharide levels was seen in all age groups, it can be presumed that the oligosaccharide excretion rather depends from the severity of the disease than from the age of the patients. In patients affected by mucopolysaccharidosis type VI a similar observation was made: MPS VI patients who had low height values excreted higher amounts of glycosaminoglycans than those who had normal or almost normal height values [29]. From these findings it can be concluded that urinary oligosaccharide excretion can be used as a surrogate marker in clinical trials for example for the development of therapeutic principles such as enzyme replacement therapy.

## Conclusions

The variable clinical presentation of the Mannosidosis patients investigated in this survey confirms that alpha-Mannosidosis is a highly heterogeneous disorder, as it has already been described by several authors [1,3,22]. It could be demonstrated that there was only slight progression of a few clinical findings within the observational time: In both groups psychiatric troubles, and impaired lung function in patients under the age of 18 years. Our study has revealed that in both age groups significant neurological abnormalities such as ataxia and dysarthria can be observed. And in a mouse model for alpha-Mannosidosis it could be demonstrated that the intravenous administration of recombinant alpha-Mannosidase resulted in an improvement of the neuromotor disabilities found in untreated mice [30]. From these results it can be considered to incorporate not only the lung function and 6MWT or 3stair climb test, but also neurological investigations as clinical parameters for therapeutic clinical trials [31]. As additional biomarkers oligosaccharide levels in serum and/or urine may serve.

## Abbreviations

MALDI-TOF: Matrix-assisted laser desorption/ionization time-of-flight mass spectrometry; BMI: Body Mass Index; dB HL: Decibel/hearing level; VAS: Visual Analogue Scale; SD (or SE): Standard deviation.

## Competing interests

The study is funded by the EU in the “HUE-MAN” consortium through a Framework 6 grant of 3.2 million Euro for a period of 36 months. The entire grant covers both scientific programs and clinical programs. The authors; except Jens Fogh, who is employed at the Danish company Zymenex; confirms independence from any sponsors; the content of the article has not been influenced by any sponsors.

## Authors’ contributions

DM has designed the study and has evaluated patients. PS and JF are the coordinators of the project. MB, JZ and JEW have performed the clinical evaluations of the patients. JCM has performed the biochemical analyses. KJO has carried out the statistical analysis. All authors contributed to the concept of the study and participated in drafting the manuscript and approved the final manuscript.
